# A novel biallelic variant c.2219T > A p.(Leu740*) in *ADGRG6* as a cause of lethal congenital contracture syndrome 9

**DOI:** 10.1111/cge.14237

**Published:** 2022-10-09

**Authors:** Mangalore S. Shravya, Mary Mathew, Akhila Vasudeva, Katta M. Girisha, Shalini S. Nayak

**Affiliations:** 1 Department of Medical Genetics, Kasturba Medical College, Manipal, Manipal Academy of Higher Education, Manipal, India; 2 Department of Pathology, Kasturba Medical College, Manipal, Manipal, Academy of Higher Education, Manipal, India; 3 Department of Obstetrics and Gynaecology, Kasturba Medical College, Manipal, Manipal Academy of Higher Education, Manipal, India

## Dear Editor

Adhesion G protein-coupled receptor G6 (ADGRG6) is a major constituent of the basement membrane essential for normal myelination of axons. To date, variants in *ADGRG6* are known to be associated with lethal congenital contracture syndrome 9 (LCCS9; MIM#616503) in three published reports comprising of six families and additionally, two families with intellectual disability and joint contractures.^1–5^ Here, we describe an additional Asian family with a novel variant in *ADGRG6* as a cause of LCCS9.

A consanguineous couple had an intrauterine fetal demise at 24 weeks of gestation following preeclampsia in their first conception. In the second pregnancy, ultrasonography at 18 weeks of gestation showed a short and curved left thumb, flexion deformity at the left wrist, and early onset of intrauterine fetal growth restriction. At 32 weeks of gestation polyhydramnios was noted. The family history was unremarkable. The informed consent for the study was obtained. The perinatal assessment was done following stillbirth. The female fetus weighed 1404 g (−0.957 SD), measured 33 cm (−4.2 SD) in length, and had a head circumference of 32 cm (+1.82 SD). Clinical information noted in the fetus is described in Table 1. Skeletal survey and microscopic evaluation of the biceps, femoral nerve, and diaphragm were normal.

Chromosomal microarray from fetal DNA was normal. Trioexome sequencing (TWIST Biosciences) identified, a novel stop-gain biallelic variant c.2219 T > A p.(Leu740*) in exon 15 of *ADGRG6* in the proband. Parents are heterozygous for the variant and are healthy. This variant is not observed in any individual in the gnomAD database and our in-house database of 1683 exomes. In-silico prediction tools predict this variant results in nonsense-mediated mRNA decay or in the formation of non-functional protein. The variant is classified to be pathogenic according to the ACMG guidelines.

The clinical features associated with LCCS9 are facial dysmorphism, pulmonary hypoplasia, and arthrogryposis, which are in concordance with our family.^2,3^ Notably, the Iranian family presented with intellectual disability, speech impairment, microcephaly, seizures, cerebellar hypoplasia, and spasticity with join contractures.^3^ Two siblings from other family had intellectual disability, optic atrophy, agenesis of corpus callosum, sensorimotor neuropathy, joint contractures, and cardiac valve anomaly along with biallelic variants in one more locus (*C12ORF65*), thus explaining some features (Table 1).^5^ Gpr126 is required for the normal ear development in zebrafish and in the heart, spine, and body length/mass development in mice.^1,6^ Adolescent idiopathic scoliosis has been associated with Gpr126 in mice earlier.^6^ GPR126 modulates angiogenesis through VEGF signaling and is important in cardiovascular development in mice and zebrafish.^7^ It is also known that variants in *ADGRG6/GPR126* are linked to breast and bladder cancers in humans.^6^

The *GPR126*, consists of signal peptide (SP), Complement C1r/C1s, Uegf and Bmp1 (CUB), Pentraxin (PTX), GPCR autoproteolysis-inducing (GAIN), and a C-terminal 7 transmembrane (7TM) domains. The GAIN domain is conserved and mediates an auto-catalytic cleavage crucial for activating GPCR126 signaling.^1,3^ All individuals (2/9 families) with variants (missense and truncating) in the GAIN domain are observed with neonatal deaths.^1^ Similarly, we also report a stillbirth with a biallelic stop-gain variant in the GAIN domain. Notably, few individuals (family with a missense variant in the 7TM domain and other family with a missense variant between PTX and GAIN domain) are with less severe phenotype or non-lethal and are alive up to adolescence with intellectual disability (Table 1).^2–5^ However, we do not know the reason behind the varying severity of the phenotype. In addition, we could not establish an association between the type of variant, effect on protein, and the severity of phenotype. A greater number of families will provide further insights.

To conclude, the phenotype of *ADGRG6*-related disorders ranges from lethal arthrogryposis in fetuses to intellectual disability and joint contractures in adults. Our study delineates an additional family with LCCS9 and further explores the reported families.

## Figures and Tables

**Table 1:** Clinical and molecular profile of individuals assessed in the present and previous studies

Ravenscroft et al.^1^						Laquerriere et al.^2^		Hosseini et al.^3^		Daum et al.^4^	Karaca et al.^5^		Present study
		Family 1 Individual 1	Family 2 Individual 1	Family 3		Family 4 Individual 1	Family 5 Individual 1	Family 6		Family 7 Individual 1	Family 8^a^		Family 9 Individual 1
				Individual 1	Individual 2			Individual 1	Individual 2		Individual 1	Individual 2	
Gestation/age		20 weeks	Neonate	36 weeks	19 weeks	NA	NA	16 years	19 years	NA	NA	NA	32 weeks
Consanguinity		Yes	Yes	Yes	Yes	Yes	Yes	Yes		Yes	Yes		Yes
Gender		NA	Female	Female	Male	Male	Male	Male	Male	Female	Male	Female	Female
Clinical features	Facial dysmorphism	No	Ocular hypertelorism, micrognathia	Triangular face, depressed nasal bridge, anteverted nares, thin upper lip, micrognathia, low set ears	Micrognathia, low set ears	No	No	Depressed nasal bridge, thin upper lip, low-set ears, strabismus, hypertelorism, smiling face, and widely spaced teeth		NA	Deeply set eyes, short palpebral fissures, exotropia		Long forehead, telecanthus, depressed nasal bridge, anteverted nares, long philtrum, low set ears
	Limbs	Distal joint contractures, including talus valgus, wrist and finger flexion, pterygium of right elbow and axilla	Upper-limb arthrogryposis with ulnar deviation of the hands, camptodactyly, sparse dermal ridges, knee-joint ankylosis, talipes equinovarus	Abducted thumbs, ulnar deviation, fixed flexion contractures of the hands and wrists, reduction of digital creases, thoracic kyphoscoliosis, bilateral severe talipes equinovarus, generally reduced muscle bulk in the limbs	Bilateral severe talipes equinovarus, restricted movement of the knees and shoulders, extension contractures of the elbows, flexion contractures of wrists and fingers	Joint contractures in two or more body areas during pregnancy	Joint contractures in two or more body areas during pregnancy	Paraplegia, spasticity in the upper and lower extremities associated with joint contracture, muscle atrophy		Arthrogryposis (club hand, claw hands)	Contractures of limbs		Bilateral contractures across the elbows and wrist, clenched fist on left hand, medial and lateral deviation of the fingers, camptodactyly of right-hand digits, proximally placed right thumb, bilateral contractures across the knee, rocker bottom feet
	Other	Diaphragmatic defect	No	Pulmonary hypoplasia, short umbilical cord	No	No	No	Seizures, cerebellar hypoplasia, widened fourth ventricle	Seizures	No	Intellectual disability, optic atrophy, agenesis of the corpus callosum, sensorimotor neuropathy, cardiac valve anomaly		Enlarged thymus, pleural effusion, ascites with hypoplasia of both lungs
Outcome		Termination of pregnancy	Neonatal death	Neonatal death	Termination of pregnancy	Normal delivery (further information not available)	Termination of pregnancy	Alive	Death at 19 years (unknown reason)	NA^b^	Alive	Alive	Stillbirth
*ADGRG6 (GPR126);* NM_198569.3													
cDNA and protein change		c.19C > T (exon 2)	c.2144dup (exon 15)	c.2306T > A (exon 16)		c.3601T > C (exon 25)	c.19C > T (exon 2)	c.3264G > C (exon 22)		c.3264G > A (exon 22)	c.1880A > G (exon 13)		c.2219T > A (exon 15)
		Hom; p.(Arg7*)	Hom; p. (Gln716Thrfs*16)	Hom; p.(Val769Glu)		Hom; p. (Ser1201Pro))	Hom; p.(Arg7*)	Hom; p.(Trp1088Cys)		Hom; p. (Trpl088*)	Hom; p.(Lys627Arg)		Hom; p.(Leu740*)
Type of variant		Stop gain	Frameshift	Missense		Missense	Stop gain	Missense		Stop gain	Missense		Stop gain
Protein domain		Signal peptide	GAIN	GAIN		C terminal	Signal Peptide	7TM		7TM	Between PTX and GAIN		GAIN
Predicted effect on the protein		NMD	NMD	Change in the amino acid sequence		Change in the amino acid sequence	NMD	Change in the amino acid sequence		NMD	Change in the amino acid sequence		NMD

*Note:* Asterisk indicates the termination codon.

Abbreviations: 7TM, 7-pass transmembrane domain; GAIN, GPCR autoproteolysis-inducing domain; Hom, homozygous; IUGR, intrauterine growth retardation; NA, not available; NMD, nonsense mediated decay; PTX, pentraxin domain.

a The patients also have biallelic variants in C12ORF65 known to cause spastic paraplegia 55.

b Previous two children died shortly after birth, with IUGR, oligohydramnios, club feet and claw hands.

## Data Availability

Available from the corresponding author upon reasonable request.
